# Bainbridge-Ropers Syndrome in a Texan Boy: A Case Report and Review of the Literature

**DOI:** 10.7759/cureus.32902

**Published:** 2022-12-24

**Authors:** Tania Siu Xiao, Giuliana Colombari Arce, Andreina Rojas Marron, Guadalupe A Benitez, Rebecca Schwanecke

**Affiliations:** 1 Research, Larkin Community Hospital, Miami, USA; 2 Research, Universidad de Ciencias Médicas, San Jose, CRI; 3 Pediatrics and Neonatology, Baylor College of Medicine, Houston, USA

**Keywords:** autosomal dominant, congenital renal dysplasia, asxl3-related disorder, brps, bainbridge-ropers syndrome

## Abstract

Bainbridge-Ropers syndrome (BRPS) or additional sex combs-like 3 (ASXL3)-related disorder is a neurodevelopmental disorder caused by a de novo missense mutation in the ASXL3 gene found on chromosome 18. The number of BRPS cases recorded to date is less than 100. In this report, a six-year-old Texan boy with global developmental delay, aggressive behavior, insomnia, microcephaly, strabismus, facial dysmorphic features, vesicoureteral reflux (VUR), bilateral congenital renal dysplasia, gastroesophageal reflux disease (GERD), hypotonia, failure to thrive, dysphagia, and status post-gastrostomy tube was referred to Children's Health in Dallas for evaluation. The patient shares a chromosomal abnormality with his father that did not explain his clinical findings. Therefore, further tests were indicated and a whole-exome gene sequencing revealed a de novo pathogenic heterozygous mutation in the ASXL3 gene in chromosome 18q12.1 associated with autosomal dominant BRPS. To our knowledge, this is the first case of BRPS with bilateral congenital renal dysplasia and may be correlated to the presence of the ASXL3 gene in renal tissue. This discovery provides significant new information about this condition that might be essential for comprehending it.

## Introduction

Bainbridge-Ropers syndrome (BRPS) or additional sex combs-like 3 (ASXL3)-related disorder is a neurodevelopmental disorder caused by a de novo truncating or missense mutation in the ASXL3 gene found on chromosome 18q12.1 [1-4]. BRPS is currently listed in the Online Mendelian Inheritance in Man (OMIM) database as OMIM #615485. It was first described in 2013 by Bainbridge et al. as a heterozygous pathogenic variant in ASXL3 proband [1,2,5]. Mutations in the ASXL family have been related to neurological disorders, malignancy, and congenital heart disease [1,2,4]. Although symptoms vary among patients and the illness typically manifests early, studies have found that 100% of patients have a speech delay and 99% have an intellectual disability [2]. Other significant findings include feeding problems, hypotonia, growth retardation, autism, sleep disturbance, skeletal deformities (marfanoid habitus), and abnormal facial features (prominent forehead, high-arched palate, thin eyebrows, hypertelorism, downslanting palpebral fissures, long nose with broad tip and prominent nasal bridge, and wide mouth with everted lower lip) [1,2,4,6,7]. Prevalence is not well known and to date only around 100 cases have been reported [2,3]. Diagnosis is made once there is proband identification of the mutation by molecular genetic testing, mainly by whole-exome sequencing [1,2]. Treatment and management options seek to alleviate the symptoms caused by the syndrome's clinical manifestations [2,4,8]. This case study aims to provide relevant data about a patient with BRPS, a rare and recently identified genetic disorder.

## Case presentation

A six-year-old Texan boy with global developmental delay, aggressive behavior, insomnia, microcephaly, strabismus, facial dysmorphic features, vesicoureteral reflux (VUR), bilateral congenital renal dysplasia, gastroesophageal reflux disease (GERD), hypotonia, failure to thrive, dysphagia, and status post-gastrostomy tube was referred to Children's Health in Dallas for evaluation. 

The patient was born at 39 weeks by spontaneous vaginal delivery. Pregnancy was normal with regular prenatal visits and unremarkable laboratory tests. However, there was an umbilical cord rupture at the time of delivery. Although he did not have difficulty breathing, he was unable to breastfeed and was started on expressed breast milk and formula. 

At five weeks of age, the patient was admitted for the first time to the hospital and diagnosed with urinary tract infection (UTI) and VUR. Further evaluation showed bilateral congenital renal dysplasia. At seven months of age, he was admitted to the hospital for intractable vomiting (projectile in nature). He was found to have GERD and oral/pharyngeal dysphagia. At 10 months of age, he underwent G button placement with Nissen fundoplication due to continued GERD. 

**Figure 1:**
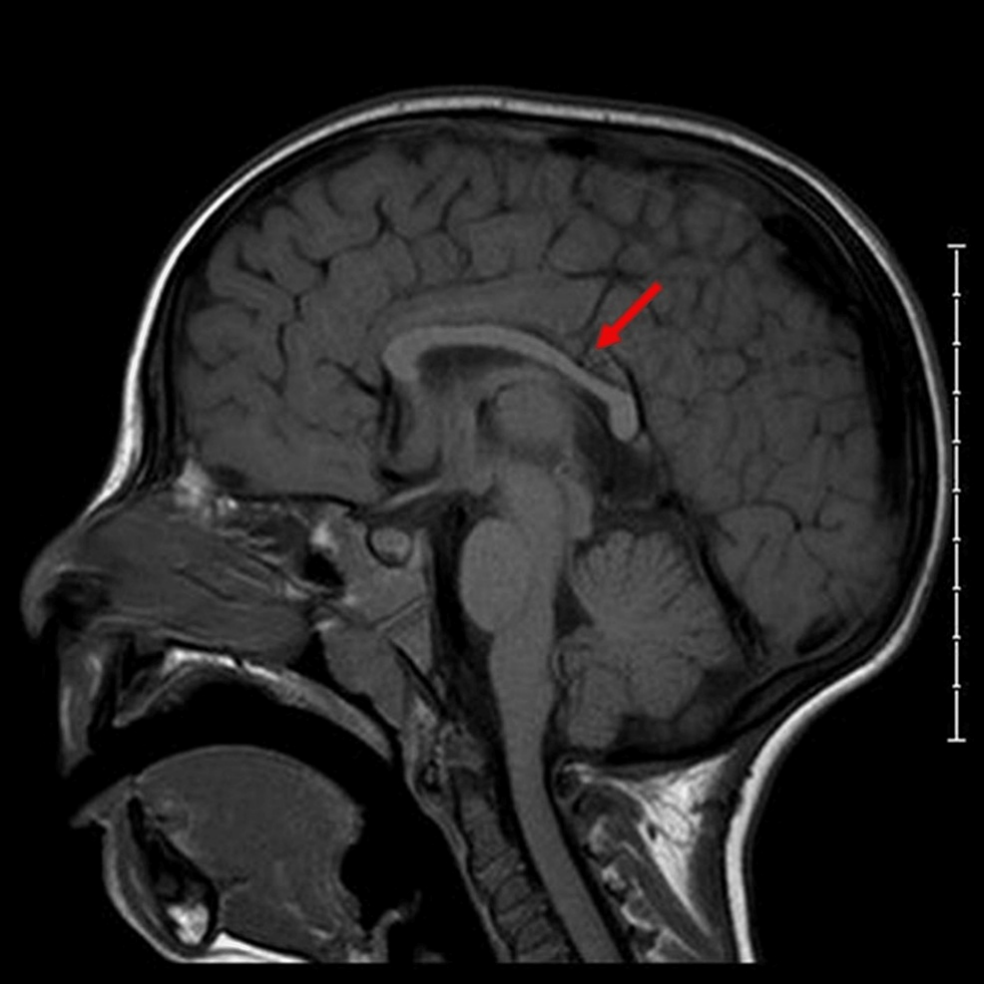
Non-contrast brain MRI. Sagittal midline T1-weighted image showing thinning of the corpus callosum (red arrow).

At three years of age, the patient underwent bilateral ureteral reimplantation due to repeated UTIs. Throughout the same time frame, he started to show symptoms of aggressive behavior with self-inflicted injury (head banging), insomnia, as well as a noticeable global developmental delay. Consequently, oxcarbazepine was indicated to manage aggressive behavior; trazodone and clonidine to manage insomnia. Additional relevant physical findings described a hypotonic infant with bilateral ptosis, hypertelorism, a hypopigmented lesion on his left neck, mongolian buttock spots, and pes planus. During the hospitalization, the patient was evaluated by neurology who recommended extensive tests, including basic metabolic screening, brain magnetic resonance imaging (MRI), and rigorous genetic tests due to severe global developmental delay and facial dysmorphic features. The patient’s non-contrast brain MRI showed thinning of the corpus callosum and no structural abnormalities (Figure 1). Chromosomal microarray analysis (CMA) revealed a gain in 1q44 copy number. The parents were also tested, and the father was found to have the same chromosomal abnormality. However, this chromosomal abnormality did not explain the patient’s findings and further tests were indicated. Additionally, a leukodystrophy panel for lysosomal enzymes as well as gene/DNA testing were unremarkable ruling out lysosomal storage disorders, trisomies, and Fragile X syndrome.

After five years of indefinite testing yielding no accurate results, a whole-exome genome sequencing was performed revealing a de novo pathogenic heterozygous mutation in the ASXL3 gene in chromosome 18q12.1 associated with autosomal dominant BRPS.

## Discussion

A mutation in the ASXL3 gene on chromosome 18q12.1 causes the exceedingly rare genetic condition known as BRPS [1-4]. It was initially identified in 2013 by Bainbridge et al. [1,2,5]. They found that some patients with clinical manifestations resembling those of Bohring-Opitz syndrome (BOS), which is associated with de novo truncating or missense mutations in the ASXL1 gene, actually had abnormalities in the ASXL3 gene rather than ASXL1 mutations [5]. 

BRPS is characterized by severe psychomotor development delay, feeding problems, hypotonia, growth retardation, intellectual disabilities, autism, sleep disturbance, skeletal deformities (marfanoid habitus), and abnormal facial features (prominent forehead, high-arched palate, thin eyebrows, hypertelorism, downslanting palpebral fissures, long nose with broad tip and prominent nasal bridge, and wide mouth with everted lower lip) [1,2,4]. However, not every clinical manifestation is present and symptoms vary among patients. Studies have found that 100% of patients have a speech delay and 99% have an intellectual disability [2]. In this case report, the patient presented with a protruding forehead, an elongated face, hypertelorism, bilateral ptosis, wide mouth, microcephaly, bilateral pes planus, hypotonia, and a global developmental delay that included motor, speech, and cognitive impairments. 

According to Balasubramanian et al., the ASXL family encodes presumptive polycomb proteins, which likely combine with other proteins to act as histone methyltransferases. By controlling the transcription of target genes, these polycomb proteins have been associated with carcinogenesis and embryogenesis [1]. Therefore, mutations in the ASXL family have been related to neurological disorders, malignancy, and congenital heart disease [1,2,4]. ASXL3 is found in tissues similar to ASXL1, including the brain, spinal cord, kidney, liver, and bone marrow [1,2,4]. This may explain the phenotypic overlap between BRPS and BOS. 

In addition to BOS, the differential diagnosis must also consider any conditions related to intellectual impairment and dysmorphic features [2,3]. Other important differential diagnosis includes Shashi-Pena syndrome (associated with a mutation in ASXL2), trisomies, Fragile X syndrome, lysosomal storage disorders, and all intellectual developmental disorders (autosomal dominant, autosomal recessive, and X-linked) [2]. Fragile X syndrome was an important differential diagnosis that was ruled out in this patient because of his similarities to that disorder [9]. BOS, Shashi-Pena syndrome, trisomies, and lysosomal storage disorders were also ruled out in this patient. 

BRPS is rarely caused by a pathogenic variant inherited from a parent, and when neither parent has an identified pathogenic variant, there is a low likelihood of sibling inheritance [2]. Although BRPS is associated with a low risk of sibling inheritance, there is a case report of two siblings with unaffected parents and thus, we should not disregard parental mosaicism [6]. Molecular genetic testing of BRPS usually starts by performing CMA according to some studies [1,2]. If non-conclusive, a whole-exome sequence array is indicated where proband identification provides the diagnosis [1,2]. In our case report, CMA revealed a gain in 1q44 copy number in the patient. The parents were also tested, and the father was found to have the same chromosomal abnormality. As reported in a study, 1q21.1-1q44 was the most amplified genomic region. One of the most prevalent mutations in hepatocellular carcinoma is chromosome 1q copy gain, which has also been related to cholangiocarcinoma [10]. Based on this, screening for malignancy is highly recommended to this patient and his father in the future. On the other hand, this chromosomal abnormality reported in the CMA was unrelated to the patient’s findings. Hence, the patient underwent a whole-exome sequence array and was found to be positive for a de novo mutation in the ASXL3 gene on chromosome 18q12.1 associated with BRPS. This test was performed on the parents as well, and the results were negative for this mutation.

Additional examination and supplementary testing, such as MRI, electroencephalogram, complete metabolic panel, and upper gastrointestinal endoscopy, are advised to determine the severity of the syndrome [2]. In this study, the patient presented with bilateral congenital renal dysplasia, which may be due to the ASXL3 gene expressed on renal tissue. Unfortunately, there was no genetic analysis done of the patient's kidney because the diagnosis of BRPS was not established by the time the patient underwent surgery. For this reason, if patients with BRPS present with renal abnormalities, we highly recommend a renal evaluation, including urinalysis, renal imaging test, and renal biopsy with genetic analysis.

According to Balasubramanian et al., MRI imaging in three patients showed non-specific findings with white matter changes and only one patient reported vermis hypoplasia [1]. Nevertheless, most patients with BRPS have normal brain MRIs [2]. Moreover, Bainbridge et al. found one patient with mild white-matter reduction, brainstem hypoplasia, dysplasia of bilateral cerebellar tonsils, and mild inferior vermis hypoplasia [5]. Another study reported cerebral sulcus widened, cortical atrophy, and corpus callosum thinning [7]. In our study, the patient’s non-contrast brain MRI showed thinning of the corpus callosum and no structural abnormalities. These findings can correlate with the patient’s clinical developmental delay [11]. Speech delay and cognitive impairment have been linked to corpus callosum thinning, as we clearly see manifested in this patient. However, we should keep in mind that thinning of the isthmus of the corpus callosum can be a normal variant incidentally observed in 22% of people due to the connection between the corpus callosum's body and splenium [12].

Treatment and management options seek to alleviate the symptoms caused by the syndrome's clinical manifestations [2,4,8]. For instance, one of the earliest manifestations that required intervention in this patient was GERD, for which he received anti-reflux medication, and consequently Nissen fundoplication and gastrostomy. We discovered from reviewing further publications that other patients with this syndrome also underwent similar treatments [4,6-8]. Furthermore, there have been no cases reported with renal malformations that required intervention. Nevertheless, our patient presented with congenital bilateral renal dysplasia requiring bilateral ureteral reimplantation [1,2,4]. The patient also received occupational treatment, physical therapy, speech therapy, and medication for aggressive behavior and insomnia.

## Conclusions

In conclusion, our study reports a de novo mutation in the ASXL3 gene on chromosome 18q12.1 associated with the clinical manifestation of BRPS in a Texan boy. It provides additional evidence for the emerging BRPS that features intellectual disability, delayed speech, autistic symptoms, hypotonia, and distinctive facial features. To our knowledge, this is the first case of BRPS with bilateral congenital renal dysplasia and may be correlated to the presence of the ASXL3 gene in renal tissue. For this reason, we highly recommend a renal evaluation in BRPS patients with renal abnormalities. Since case reports on this condition are limited, the exact spectrum is still unknown. Therefore, further studies are required to have a better understanding of this condition. As a broad recommendation, genetic counseling and periodic surveillance should be performed on every patient with BRPS.
